# The Mutual Autopsy Society of Paris

**Published:** 1879-10

**Authors:** Henry M. Lyman

**Affiliations:** Chicago


### ARTICLE IV.

### THE MUTUAL AUTOPSY SOCIETY OF PARIS. By HENRY M. LYMAN, M. D., of Chicago.

Considerable amusement was excited, a few years ago, by the announcement that a society for mutual autopsy had been formed among the savants of Paris, with a view to advancing knowledge of the structure and physiology of the brain by a correlation of intellectual characteristics with *post mortem* appearances. The whole thing was generally regarded as a scientific joke of more than ordinary magnitude. But the society appears to have been a genuine fact, and one of its members, M. Asseline, having recently deceased, his brain was carefully examined by his surviving associates, who made a full report of the result to the Anthropological Society of Paris. The following account of the matter is found in *Nature*, Aug. 14, 1879, p. 377 :

"M. Asseline died in 1878, at the age of 49. He was a republican and a materialist ; was possessed of enormous capacity for work, great faculty of mental assimilation, and an extraordinarily retentive memory ; and had a gentle, benevolent disposition, keen susceptibilities, refined taste and subtle wit. As a writer he had always displayed great learning, unusual force of style and elegance of diction, and in his intercourse with others he had been unassuming, sensitive and even timid. Yet the autopsy showed such coarseness and thickness of the convolutions that M. Broca pronounced them to be characteristic of an inferior brain. The fossa or depressions, regarded by Gratiolet as a simian character, and as a sign of cerebral inferiority, which are often found in women, and in some men of undoubted intellectual inferiority, were very much marked, especially on the left parieto-occipital. But the cranial bones were at some points so thin as to be translucent ; the cerebral depressions were deeply marked, the frontal suture was not wholly ossified, a decided degree of asymmetry was manifested in the greater prominence of the right frontal, while, moreover, the brain weighed 1,468 grams, *i.e.*, about 60 grains above the average given by M. Broca for M. Asseline's age. The apparent contradictions between the weight of the brain and the marked character of the parieto-occipital depressions, attracted much attention, and the members of the Société d'Anthropologie have been earnestly invited by M. Hovelacque, in furtherance of science, to join the Société d'Autopsie, to which anthropology is already indebted for many highly important observations. This society is forming a collection of photographs of its members, which are taken in accordance with certain fixed rules."

